# Strawberry milk-like blood in a subject with diabetic lipemia: dramatic change to transparent color after insulin therapy

**DOI:** 10.1186/s40064-016-3202-5

**Published:** 2016-09-07

**Authors:** Atsushi Obata, Shinji Kamei, Seizo Okauchi, Tomohiko Kimura, Hidenori Hirukawa, Akihito Tanabe, Tomoe Kinoshita, Kenji Kohara, Fuminori Tatsumi, Masashi Shimoda, Shuhei Nakanishi, Tomoatsu Mune, Kohei Kaku, Hideaki Kaneto

**Affiliations:** Department of Diabetes, Endocrinology and Metabolism, Kawasaki Medical School, 577 Matsushima, Kurashiki, 701-0192 Japan

**Keywords:** Chylomicronemia, Diabetic lipemia, Hypertriglycemia

## Abstract

**Introduction:**

It is known that chylomicronemia is caused by several pathologies and is classified as primary and secondary chylomicronemia. Since hypertriglycemia is associated with an increased risk of cardiovascular disease and severe pancreatitis, it is very important to make a proper diagnosis of the cause of hypertriglycemia.

**Case description:**

We herein present the case of a 40-year-old male who developed severe hypertriglycemia accompanied with acute exacerbation of type 2 diabetes mellitus. On admission, his blood glucose level was 306 mg/dl and HbA1c was 12.5 %. Moreover, serum triglyceride level was elevated up to 5661 mg/dl. When blood was drawn, it presented strawberry milk-like color. After receiving insulin treatment, he obtained good glycemic control and the serum became back to normal transparent color.

**Discussion and Evaluation:**

Insulin resistance reduces triglyceride clearance and also increases triglyceride release from adipocyte. It is known that glucose toxicity and strong insulin resistance induce inactivation of LPL, which results in chylomicronemia.

**Conclusion:**

This case report suggests that when serum triglyceride level is markedly elevated due to diabetic lipemia, it is extremely important to obtain good glycemic control.

## Background

Chylomicronemia is caused by several pathologies and can be classified as primary and secondary chylomicronemia (Yuan et al. 2007). Making a proper diagnosis of the cause of hypertriglycemia is really important as it is strongly associated with an increased risk of cardiovascular disease and severe pancreatitis. The incidence of chylomicronemia reported by the Lipid Research Program prevalence study revealed 1.79 per 10,000 individuals (less than 0.02 %) had triglyceride levels higher than 2000 mg/dl (Brunzell et al. 1982). We present herein a case of chylomicronemia accompanied by acute exacerbation of type 2 diabetes but dramatically recovered after insulin therapy. Blood color in this subject looked like strawberry milk but dramatically changed to completely normal transparent after obtaining good glycemic control with insulin therapy.

## Case presentation

**Fig. 1:**
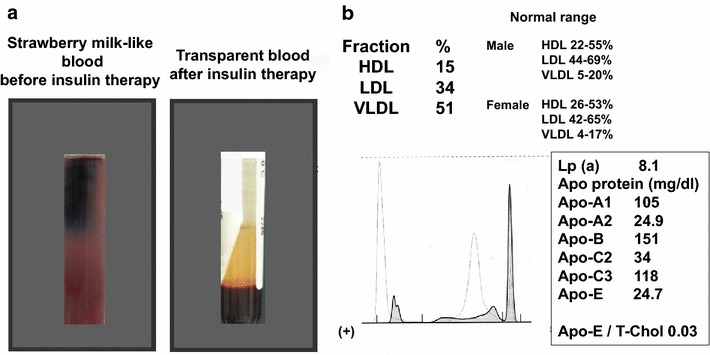
**a** Strawberry milk-like blood before insulin therapy (*left panel*). Marked change to transparent color after insulin therapy (*right panel*). Some parts of the pictures are obstructed to block personal information. **b** Treatment naive lipoprotein fraction

A 40-year-old male visited our hospital as polydipsia, polyuria and general fatigue developed approximately 2 months before. His body weight decreased from 72.7 kg (BMI 25.5 kg/m^2^) to 67.6 kg (BMI 23.8 kg/m^2^) during this term. He had been diagnosed with depression and taking multiple medications and dyslipidemia (T-Chol 263 mg/dl, LDL-C 186 mg/dl, HDL-C 48 mg/dl, TG 272 mg/dl) had been pointed out in the annual medical check-up conducted a year before. The blood drawn for examination presented strawberry milk-like appearance (Fig. 1a, left panel). Laboratory test revealed elevated fasting blood glucose and HbA1c; 306 mg/dl and 12.5 %, respectively. Furthermore, serum triglyceride was markedly elevated up to 5661 mg/dl, which presumably caused the dramatic change of blood color. He was admitted to our hospital for further examination about general fatigue, weight loss and hypertriglycemia. Serum amylase and lipase were both within normal range; 64 IU/l and 41 U/l, respectively and there were no symptoms which suggested pancreatitis. After admission, multiple daily insulin injection was started to obtain good glycemic control. The patient was diagnosed with type 2 diabetes as serum insulin level (3.4 μU/ml) and urinary C-peptide response (>200 µg/day) were preserved and autoantibodies related to type 1 diabetes were absent. No diabetic complications such as neuropathy, retinopathy and nephropathy were observed. Lipemia retinalis was not observed in funduscopy. We also conducted further examination to clarify the cause of extraordinarily elevated serum triglyceride. Chylomicronemia indicated that the patient had type V hyperlipoproteinemia. He had no habit of alcohol drinking and hypothyroidism was ruled out from the laboratory test (thyroid stimulating hormone (TSH) 1.065 μIU/ml, free T3 3.0 pg/ml, free T4 1.1 ng/dl). In addition, no medications which the patient was taking for depression have been reported to exacerbate diabetes or hypertriglycemia. We also checked LPL and ApoC-II to rule out any genetic factors just in case. As the results, ApoC-II was within normal range, which excluded ApoC-II deficiency. Serum heparinized lipoprotein lipase (LPL) was within normal range (255 ng/mL), which ruled out LDL deficiency, although LPL gene mutation screening was not conducted. In addition, we checked ApoE genotype to rule out any genetic factors just in case. As the results, his ApoE phenotype was E3/E3, which is not susceptible to hypertriglycemia compared to E2/E2 or E4/E4 (Kei et al. 2015; Shinozaki et al. 2005). Eruptive xanthomas were not observed probably because serum triglyceride was increased very rapidly together with acute exacerbation of type 2 diabetes. The result of treatment naive lipoprotein fraction was as shown in Fig. 1b, which revealed pre-β-band and prominently elevated VLDL fraction up to 51 %. This patient was taking a large amount of soft drinks due to polydipsia before admission, resulting in rapid exacerbation of type 2 diabetes and relative insufficiency of insulin action. Indeed, serum non-esterified fatty acid was extremely elevated up to 6.12 mEq/l (normal range is 0.14–0.85 mEq/l). We diagnosed this patient as diabetic lipemia as there was no finding suggesting genetic hypertriglycemia or secondary causes such as multiple myeloma, paraproteinemia and systemic lupus erythematosus.

**Fig. 2:**
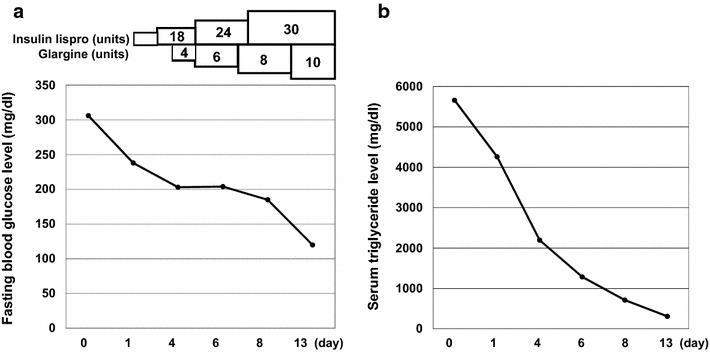
Alteration of fasting blood glucose level (**a**) and serum triglyceride level (**b**) after intensive insulin therapy

After admission, in order to obtain good glycemic control, we started multiple daily insulin injection using insulin lispro and glargine. As shown in Fig. 2a, after increasing insulin dose, blood glucose level was decreased. Finally, when we increased insulin dose up to 40 U (insulin lispro 30 U, glargine 10 U), good glycemic control was obtained; fasting blood glucose levels were approximately 100 mg/dl. As shown in Fig. 2b, serum triglyceride level was markedly decreased together with the decrease of blood glucose level. Serum triglyceride level was as high as 5661 mg/dl on admission, but its level 1, 4, 6, 8, 13 days after admission was 4261, 2196, 1287, 714, 311 mg/dl, respectively. Finally, on 14th day, when he was discharged, it was improved to 272 mg/dl and blood appearance became completely normal without using any hypolipidemic agents. As shown in Fig. 1a (right panel), after insulin therapy, the serum became transparent, and there was marked difference between before and after insulin therapy, indicating that this case was truly diabetic lipemia.

## Conclusion and discussion

Chylomicronemia is present when serum triglyceride exceeds 1000 mg/dl. Serum triglyceride levels increase as insulin resistance reduces triglyceride clearance and also increases triglyceride release from adipocyte (Brunzell et al. 1982). In this report, extremely elevated non-esterified fatty acid strongly suggests the existence of strong insulin resistance and increased triglyceride release from adipose tissue. Chylomicrone is mainly metabolized by LPL, which is activated by insulin signaling. It is known that glucose toxicity and strong insulin resistance induce inactivation of LPL, which results in chylomicronemia (Sparks et al. 2012). Exacerbation of insulin resistance in this case might have been partially attributed to prominent fatty liver in addition to glucose toxicity. As the incidence of overweight, obesity and type 2 diabetes is increasing drastically worldwide, it is very likely that chylomicronemia and chylomicronemia syndrome, which is accompanied by at least one of eruptive xanthoma, lipemia retinalis or abdominal findings such as abdominal pain, acute pancreatitis or hepatosplenomegaly, would become more prevalent in the near future. In this case, prominent chylomicronemia syndrome was not observed. However, we cannot completely exclude the possibility that we missed lipemia retinalis as funduscopy was carried out after starting insulin treatment.

It is known that the development of chylomicronemia is influenced by various genetic factors as well as the alteration of dietary life such as overeating. Indeed, his mother had dyslipidemia in her fifties, although its details were unknown. Therefore, we cannot deny the possibility that some genetic factors in addition to the acute exacerbation of type 2 diabetes mellitus are involved in the development of chylomicronemia in this subject.

In this case, serum TG level was markedly increased up to 5661 mg/dl and strawberry milk-like blood color was dramatically changed to normal transparent color after obtaining good glycemic control with insulin therapy. We think that, in everyday clinical practice, it is not so common to see subjects with type 2 diabetes accompanied by such marked increase of TG level and dramatic change of blood color. Therefore, we think that this report would call for attention from the clinical point of view. In addition, this case report strongly suggest that when serum triglyceride level is markedly elevated due to diabetic lipemia, it is very important to obtain good glycemic control in subjects with type 2 diabetes.

Although diabetes mellitus is the most common secondary cause of chylomicronemia, when it is observed, we need to seek various causes by screening lipoprotein fraction and LPL activity. Recently developed novel technology can detect monogenic dyslipidemia and may help to diagnose genetic hypertriglycemia when the treatment is not successful (Hegele et al. 2015).

Taken together, we should be aware of the possibility that chylomicronemia could be induced by acute exacerbation of glycemic control. In addition, we should keep in mind that when serum triglyceride level is markedly elevated due to diabetic lipemia, it is very important to obtain good glycemic control.

## Abbreviations

TG: triglyceride
BMI: body mass index
TSH: thyroid stimulating hormone
LPL: lipoprotein lipase
VLDL: very low-density lipoprotein

## References

[CR1] Brunzell JD, Bierman EL (1982). Chylomicronemia syndrome. Interaction of genetic and acquired hypertriglyceridemia. Med Clin North Am.

[CR2] Hegele RA, Ban MR, Cao H (2015). Targeted next-generation sequencing in monogenic dyslipidemias. Curr Opin Lipidol.

[CR3] Kei A, Miltiadous G, Bairaktari E (2015). Dysbetalipoproteinemia: two cases report and a diagnostic algorithm. World J Clin Cases..

[CR4] Shinozaki S, Itabashi N, Rokkaku K (2005). Diabetic lipemia with eruptive xanthomatosis in a lean young female with apolipoprotein E4/4. Diabetes Res Clin Pract.

[CR5] Sparks JD, Sparks CE (2012). Selective hepatic insulin resistance, VLDL overproduction, and hypertriglyceridemia. Arterioscler Thromb Vasc Biol.

[CR6] Yuan G, Al-Shali KZ (2007). Hypertriglyceridemia: its etiology, effects and treatment. CMAJ.

